# Impact assessment of immunization and the COVID-19 pandemic on varicella across Europe using digital epidemiology methods: A descriptive study

**DOI:** 10.1371/journal.pone.0283465

**Published:** 2023-04-12

**Authors:** Ugne Sabale, Ligita Jarmale, Janice Murtagh, Manjiri Pawaskar, Goran Bencina

**Affiliations:** 1 Center for Observational and Real-World Evidence (CORE), MSD, Stockholm, Sweden; 2 Marketing and Digital Operations Baltics, MSD, Vilnius, Lithuania; 3 Medical Affairs Vaccines, MSD, Dublin, Ireland; 4 Center for Observational and Real-World Evidence (CORE), Merck & Co., Inc., Rahway, NJ, United States of America; 5 Center for Observational and Real-World Evidence (CORE), MSD, Madrid, Spain; Regional Health Care and Social Agency of Lodi, ITALY

## Abstract

**Background:**

Varicella is usually a mild disease in children but may be life-threatening, especially in adolescents and adults. Infection control measures implemented during the Coronavirus Disease 2019 (COVID-19) pandemic may have suppressed varicella transmission, potentially creating an ‘immunity debt’, particularly in countries without universal varicella vaccination.

**Objectives:**

To assess trends in Google search engine queries for varicella keywords as a proxy for varicella infection rates and to evaluate the effect of universal varicella vaccination on these trends. A further objective was to assess the impact of the COVID-19 pandemic on varicella keyword search query trends in countries with and without universal varicella vaccination.

**Methods:**

This study used the keyword research tool, Google Trends, to evaluate trends in time series of the relative search query popularity of language-specific varicella keywords in 28 European countries from January 2015 through December 2021. The Google Ads Keyword Planner tool was used to evaluate absolute search volumes from March 2018 through December 2021.

**Results:**

The relative search query popularity of varicella keywords displayed marked seasonal variation. In all 28 countries, the relative search query popularity of varicella keywords declined after the start of the COVID-19 pandemic (March 2020), compared with pre-pandemic levels (range, -18% to -70%). From April 2020 to July 2021, a period of intense COVID-19 transmission and infection control, absolute search volumes for varicella keywords were lower than pre-pandemic levels but rebounded after July 2021, when infection control measures were relaxed.

**Conclusion:**

This evaluation of search query trends demonstrated that search query data could be used as a proxy for trends in varicella infection rates and revealed that transmission of varicella may have been suppressed during the COVID-19 pandemic. Consideration should be given to using search query data to better understand the burden of varicella, particularly in countries where surveillance systems are inadequate.

## Introduction

Varicella, commonly referred to as chickenpox, is a highly transmissible infection caused by the varicella-zoster virus. Varicella is usually a mild and self-limiting disease; however, it can lead to serious or fatal outcomes, even in young children, but particularly in newborns, pregnant women, adolescents, adults, and individuals with a weakened immune system [1–3]. Studies from Europe show that in the absence of vaccination, over 90% of children are infected before 12 years of age [1, 4]. The risk of severe outcomes rises steadily with age among individuals infected during adolescence or adulthood [3].

However, varicella is a vaccine-preventable disease, as vaccines have been shown to have 98% effectiveness against moderate to severe varicella [5]. Long-term evaluations of varicella outcomes in countries with universal varicella vaccination (UVV), such as Spain, Greece, Germany and Italy, indicate that UVV has led to marked declines in varicella incidence and varicella-associated hospitalizations [6, 7]. In line with these findings, European Union and World Health Organization guidance recommends that national health authorities plan for varicella vaccination programs by assessing the public health burden of varicella and the country’s capacity to sustain vaccination coverage. Where feasible and appropriate, countries should implement routine varicella immunization of at least 80% coverage using a 1- or 2-dose regimen starting at 12 to 18 months of age [8, 9]. Despite these recommendations and evidence that vaccination reduces the burden of varicella, UVV has not been widely implemented in Europe [4].

Although often clinically mild, varicella poses a substantial caregiver and economic burden [8]. However, inadequate varicella surveillance has led to considerable under-reporting of the disease burden, particularly as varicella is not on the European Union list of mandatorily reportable infections and is often not medically attended [10]. Inadequate surveillance may thus contribute to the low priority accorded to varicella vaccination in this region [7, 8], while hindering detection of perturbations in varicella transmission dynamics, such as rebounds in varicella incidence [7, 8, 11, 12].

Data suggest that infection control measures implemented during the Coronavirus Disease 2019 (COVID-19) pandemic led to altered seasonal variation and marked decreases in incidence for a number of pediatric infections [13, 14]. However, few studies have evaluated the effects of the COVID-19 pandemic on varicella incidence [12, 13], and to our knowledge, no study has assessed how these effects may vary in countries with and without UVV.

Given the limitations and expense of conventional surveillance, interest has grown in the use of digital epidemiology, such as analysis of internet search query data to evaluate patterns of disease, as both patients and clinicians increasingly rely on the internet as a health information resource [15]. Therefore, this study’s objective was to assess trends in internet keyword search queries for varicella as a proxy for trends in varicella infection rates and evaluate the effect of UVV on these trends from 2015–2021. To address the information gap regarding the impact of the COVID-19 pandemic on varicella infection dynamics, a further objective of this study was to assess trends in internet varicella keyword queries in European countries with and without UVV over a 7-year period, before and during the pandemic.

## Methods

### Search query data

Google Trends [16] and Google Ads Keyword Planner [17] are free web-based publicly accessible keyword research tools that analyze Google search queries and generate data on search patterns based on specified keywords across regions and languages. Language-specific search query data were used to evaluate trends in varicella keyword search queries and use it as a proxy informing trends in varicella infection rates. Google Trends has previously been used to evaluate trends in the incidence of pediatric infections and was found to accurately predict the epidemiologic patterns [18–20]. Google Trends issues data on relative search query popularity using the Google Trends Index, which is calculated as the number of searches for a specific keyword divided by all searches within a defined geographic area and timeframe. This index is standardized to range from 0 to 100, where 100 represents the peak popularity of the keyword during the time series, excluding repeated searches conducted by the same user during a short time period. Data on absolute search query volumes were generated using the Google Ads Keyword Planner, which quantifies the average number of keyword search queries conducted in the Google search engine in a selected geographic area and timeframe, providing up to 4 years of data.

Google Trends analyses were conducted from January 1, 2015, through December 31, 2021, and Google Ads Keyword Planner analyses were conducted from March 1, 2018, through December 31, 2021. Study periods were selected to optimize evaluation of varicella seasonal variation, the effects of UVV introduction, and the impact of the COVID-19 pandemic on varicella transmission dynamics.

For each country or region included in the study, 2 datasets were generated, one for monthly relative search query popularity (Google Trends) and another for absolute search query volume (Google Ads Keyword Planner). Datasets were extracted and transferred into Excel 2010 (Microsoft Corporation) for validation, storage, analysis, and visualization. To validate the study data, a second reviewer independently extracted and compared data tables with those from the first extraction. Search volume data could not be associated with users’ identity, IP address, or precise physical location.

### Study sample and keyword search terms

The study analysis comprised 28 European countries, including 7 that have implemented UVV (Finland, Latvia, Germany, Italy, Spain, Hungary, and Greece), 4 of which did so during the study period (Hungary, Italy, Finland, and Spain; Table 1). Local language keywords for varicella in each country were defined and validated by quantifying the search hits. In countries with more than 1 official language, keywords were identified for each language (Table 1). Uniform resource locator (URL) information for keywords searches used to generate study data were documented (S1 Table).

**Table 1:** Varicella vaccination and varicella keyword search terms for the 28 European countries included in the study.

		UVV introduction	Dosing schedule	VCR
**UVV recommended and funded**				
Finland	Vesirokko	2017 (September)	dose 1: 18 mo-11 y	83% (dose 1)
			dose 2: 6 or 12 y	
Germany	Windpocken	2004, 2009^A^	dose 1: 11–14 mo	89% (dose 1)
			dose 2: 4 wk after dose 1	85% (dose 2)
Greece	ανεμοβλογιά	2008 (January)	dose 1: 12–15 mo	95% (dose 1)
			dose 2: 2–3 y	90% (dose 2)
Hungary	Bárányhimlő	2019 (September)	dose 1: 13 mo	>90%^B^
			dose 2: 16 mo	
Italy	Varicella	2017	dose 1: 13–15 mo	90%^C^ (dose 1)
			dose 2: 5–6 y	
Latvia	Vējbakas	2009 (January)	dose 1: 12–15 mo	97% (dose 1)
			dose 2: 7 y	
Spain	Varicela	2016	dose 1: 15 mo	95% (dose 1)
			dose 2: 3–4 y	84% (dose 2)
**Vaccination recommended for at-risk groups, with or without funding**				
Austria	Windpocken			
Belgium	Windpokken, Varicelle			
Croatia	Vodene kozice			
Poland	Ospa			
Switzerland	Windpocken, Varizellen			
**No recommendation for varicella vaccination**				
Bulgaria	Вaрицела			
Czech Republic	Neštovice			
Denmark	Skoldkopper			
Estonia	Tuulerõuged			
France	Varicelle			
Ireland	Chickenpox			
Lithuania	Vėjaraupiai			
Netherlands	Waterpokken			
Norway	Vannkopper			
Portugal	Varicela			
Romania	Varicelă			
Serbia	Ovčije boginje			
Slovakia	Ovčie kiahne			
Slovenia	Norice			
Sweden	Vattkoppor			
United Kingdom	Chickenpox			

UVV, universal varicella vaccination; VCR, vaccination coverage rate

^A^ One-dose UVV introduced in 2004; 2-dose UVV introduced in 2009.

^B^ Thirty-five percent before the introduction of UVV.

^C^ The VCR in 2020.

### Statistical analysis

Time series graphs of relative and absolute monthly varicella keyword search queries in each country were visually evaluated for seasonal variation and long-term trends. Long-term trends were assessed for each country individually as well as by country clusters based on UVV status (with UVV vs. without UVV). In countries with more than 1 national language, data were evaluated within each lingual region (e.g., Wallonia and Flanders in Belgium). Differences in the relative search query popularity of varicella keywords before and after the introduction of UVV were assessed.

To evaluate the effect of the COVID-19 pandemic on search query trends, graphs were developed showing the percentage change in absolute query volumes during the first 3 full months of the pandemic (April, May, and June 2020) compared with the last month of the pre-pandemic period (March 2020). March was considered a pre-pandemic month, as the World Health Organization declared the COVID-19 pandemic on March 11, 2020 [21]. Graphs were also developed to evaluate the percentage change in absolute query volumes just before and during the pandemic (March 2020 through December 2021) compared with 2-year average volumes during the same months in the pre-pandemic years of 2018 and 2019. To visually assess the effect of the COVID-19 pandemic on search query activity, time-trend graphs were developed for countries with no UVV, which plotted monthly relative search query volumes against values predicted in the absence of the pandemic. Predicted values were estimated by averaging Google Index volumes from the same calendar months of the 5-year period preceding the COVID-19 pandemic (i.e., 2015 through 2019).

### Validation of relative search query popularity as a proxy for varicella incidence

To assess whether trends in relative search query popularity are a reasonable proxy for varicella infection dynamics, we evaluated data from Bulgaria, which was selected for validation as varicella is a mandatorily reportable disease in Bulgaria where high-quality national data on varicella cases are available [22]. A graph of Google Trends data was overlaid with monthly totals of reported cases of incident varicella for Bulgaria for the period of January 2015 through December 2021. The weekly number of cases reported by the Bulgarian National Center of Infectious and Parasitic diseases were summed to estimate the monthly number of varicella cases [22]. To quantitatively evaluate the validation dataset from Bulgaria, we developed a univariate linear model, which regressed reported varicella cases against relative search query volumes. We further validated our analytic approach by empirically evaluating the correspondence between study data and monthly trends in varicella incidence and varicella-associated hospitalizations in published reports from Croatia [23], Sweden [24] and Italy [25].

## Results

### Seasonal variation in the relative search query popularity of varicella keywords

**Fig 1:**
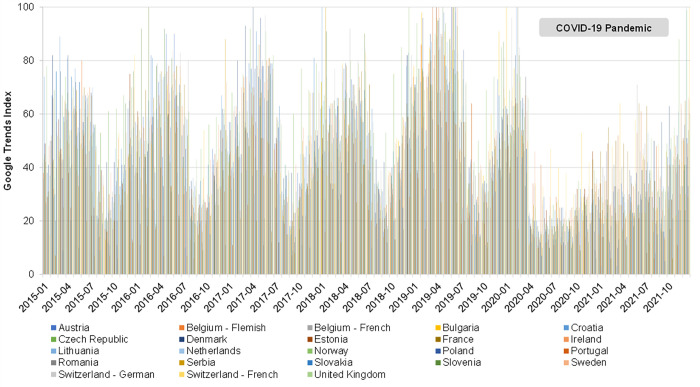
Monthly relative search query popularity of varicella keywords in European countries without universal varicella vaccination, 2015–2021. The Google Trends Index is calculated as the number of keyword searches divided by the total number of searches within a specific location and time period; it is standardized to range from 0 (no search activity) to 100 (peak search activity).

In the 5 years preceding the COVID-19 pandemic, 2015 through 2019, the relative search query popularity of varicella keywords displayed pronounced seasonal variation in most countries without UVV (Fig 1). Seasonal peaks in relative search query popularity occurred in the autumn or winter (October to January) and in the spring (March to June). A similar seasonal pattern was observed for the absolute volume of varicella keyword searches in European countries without UVV (S1 Fig).

### The effect of universal varicella vaccination on trends in the relative search query popularity of varicella keywords

**Fig 2:**
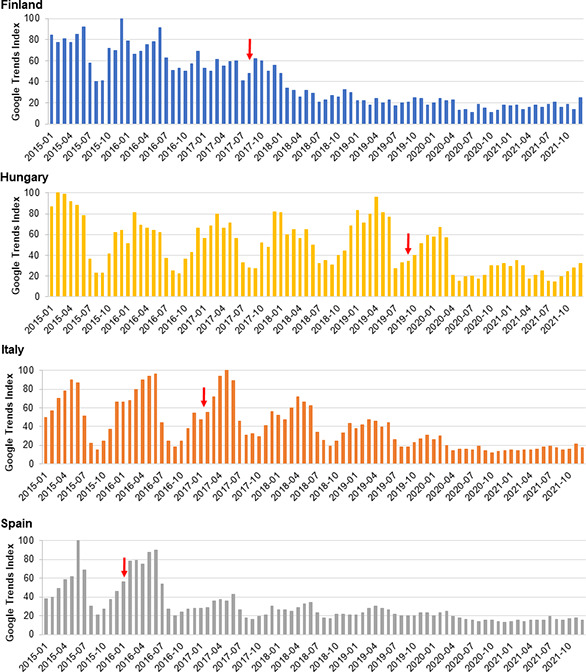
Monthly relative search query popularity of varicella keywords, 2015 to 2021, in four countries with UVV: Finland, Hungary, Italy, and Spain. The Google Trends Index reflects the relative search query popularity. The red arrow indicates when UVV was introduced into the country.

Time series graphs of the relative search query popularity of varicella keywords displayed altered seasonal variation after the introduction of UVV. UVV was introduced in Spain in 2016 and in Italy and Finland in 2017, before which the relative search query popularity of varicella keywords had pronounced seasonal variation, like that of other countries without UVV. However, after UVV was introduced, both the magnitude and the seasonal variation of varicella search queries in these 3 countries gradually diminished (Fig 2). Similarly, in Hungary, the introduction of UVV in 2019 was followed by a decline in the magnitude and the seasonal variation of the relative search query popularity of varicella keywords, albeit to a lesser extent than observed in Spain, Italy, and Finland. In Germany, Greece, and Latvia, where UVV was introduced several years before the study period, the relative search query popularity of varicella keywords displayed little seasonal variation compared with European countries without UVV (S2 Fig).

### Validation of search query popularity as a proxy for incident cases of clinical varicella

**Fig 3:**
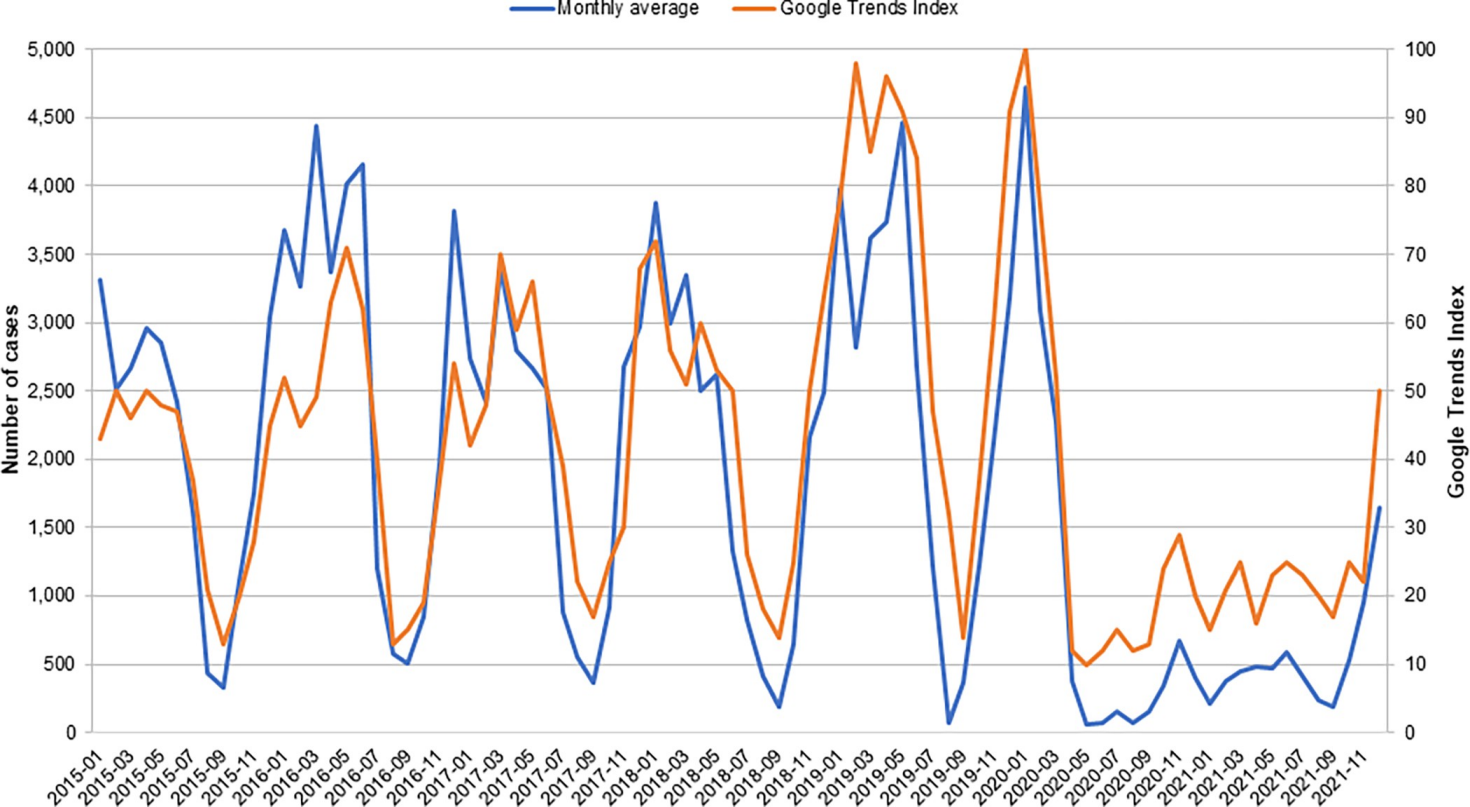
Relative search query popularity of varicella keywords and varicella cases in Bulgaria, January 2015 to December 2021. Monthly varicella case data are based on aggregated weekly case counts reported by the National Center of Infectious and Parasitic Diseases in Bulgaria [22]. The blue line shows these data converted to monthly averages, and the orange line is the Google Trends Index for internet searches performed in each month.

A validation analysis using data from Bulgaria showed a close relationship between the relative search query popularity of varicella keywords and incident cases of clinical varicella (Fig 3). Furthermore, in univariate modeling, reported varicella corresponded well with values predicted using Google Trends data; the model’s adjusted R^2^ was 0.75, indicating that Google Trends data account for a sizable proportion of the variability of reported case data (S3 Fig). Data on relative search query popularity for varicella keywords also corresponded closely to published reports of monthly varicella-associated hospitalization rates in Italy [25] and monthly varicella incidence in Croatia [23] and Sweden [24].

### The effect of the COVID-19 pandemic on the volume of search queries for varicella keywords

**Fig 4:**
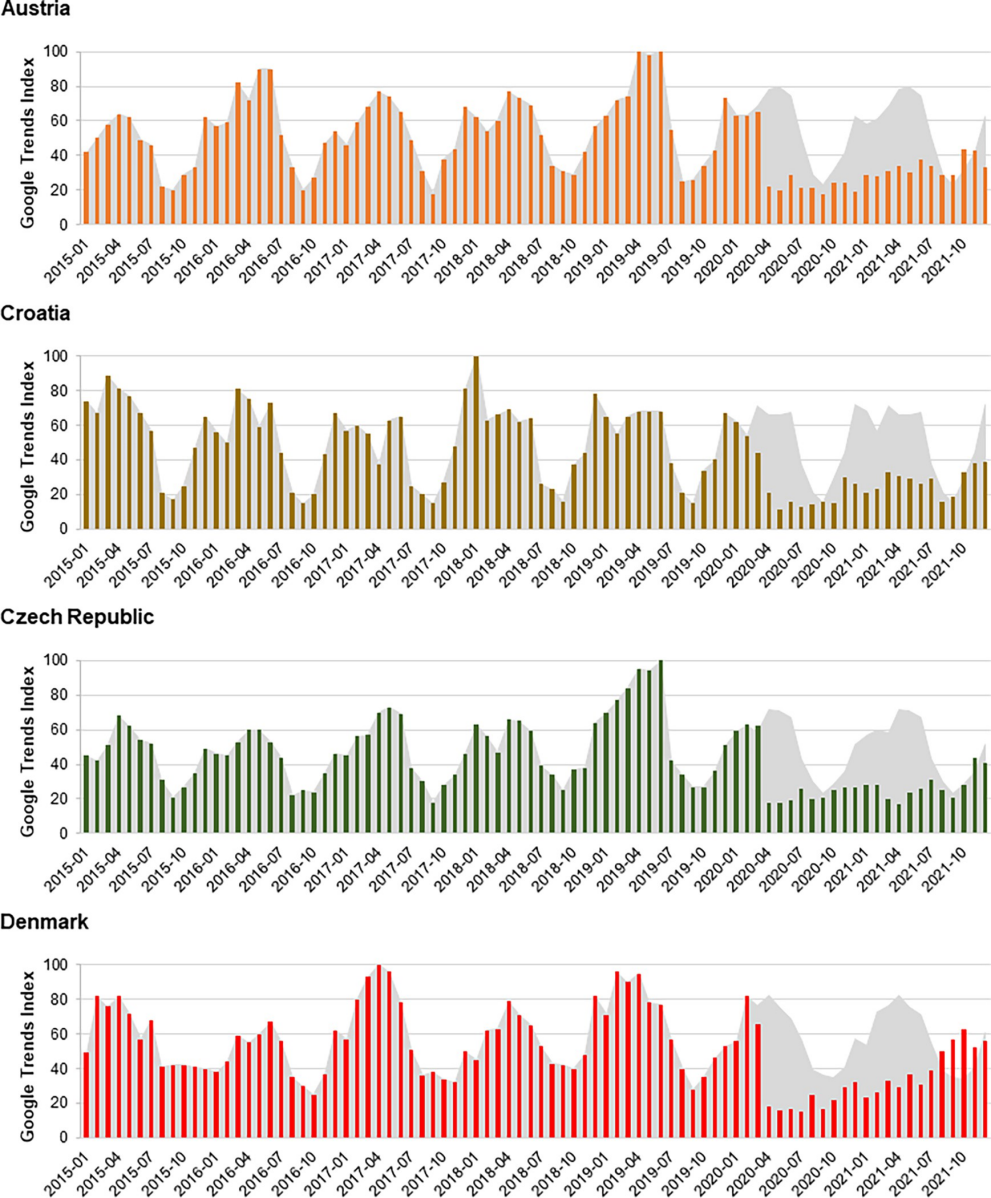
Monthly relative search query popularity of varicella keywords, 2015 to 2021, in four countries without UVV: Austria, Croatia, Czech Republic, and Denmark. The Google Trends Index reflects the relative search query popularity. The gray area shows the search query popularity predicted in the absence of COVID-19, estimated as the 5-year average of Google Index values observed in the same calendar month, from 2015 through 2019.

**Fig 5:**
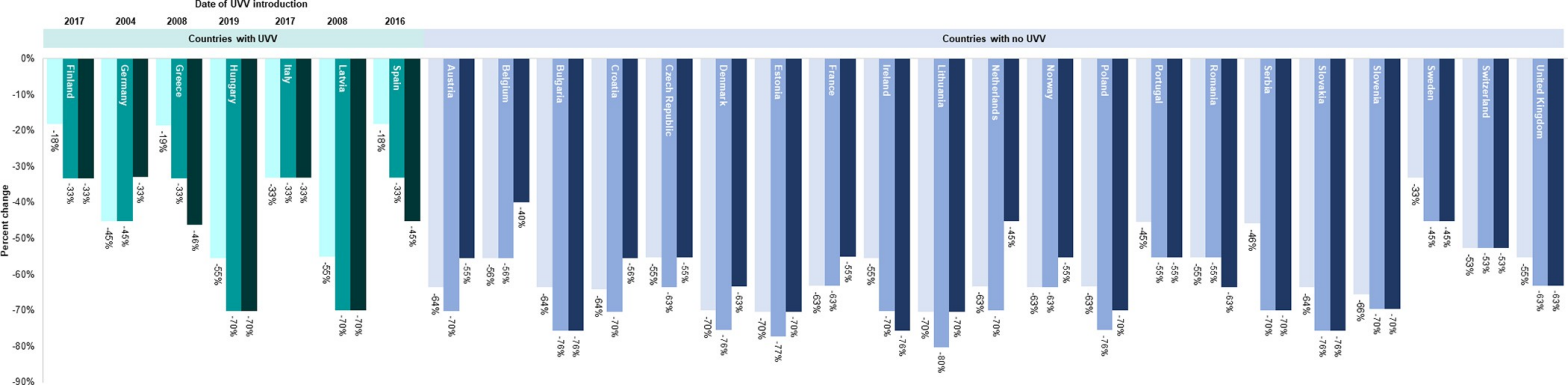
Percentage change in absolute search query volume from March 2020, the start of the COVID-19 pandemic, to April, May, and June 2020. Bars represents the percent decline in search query volume from March to April 2020 (lightest bar), March to May 2020 (medium bar), and March to June 2020 (darkest bar).

In countries with no UVV, relative search query volumes were markedly lower during the pandemic period compared with values predicted in the absence of the pandemic (Fig 4; S1 Fig). Similarly, COVID-19 appeared to have a downward effect on trends in absolute search query volumes. In the European region, seasonal peaks in the absolute volume of search queries for varicella keywords usually include the month of March, a pattern observed in 2020 in almost all the study countries (S4 Fig). However, all 28 countries included in the study recorded abrupt and unseasonal declines in the absolute volume of search queries for varicella keywords during April, May, and June 2020, the first 3 months of the COVID-19 pandemic (Fig 5). These declines were more pronounced in countries without UVV, and in most countries intensified over time, with March to June declines ranging from 33% to 70% in countries with UVV and from 40% to 76% in countries without UVV.

**Fig 6:**
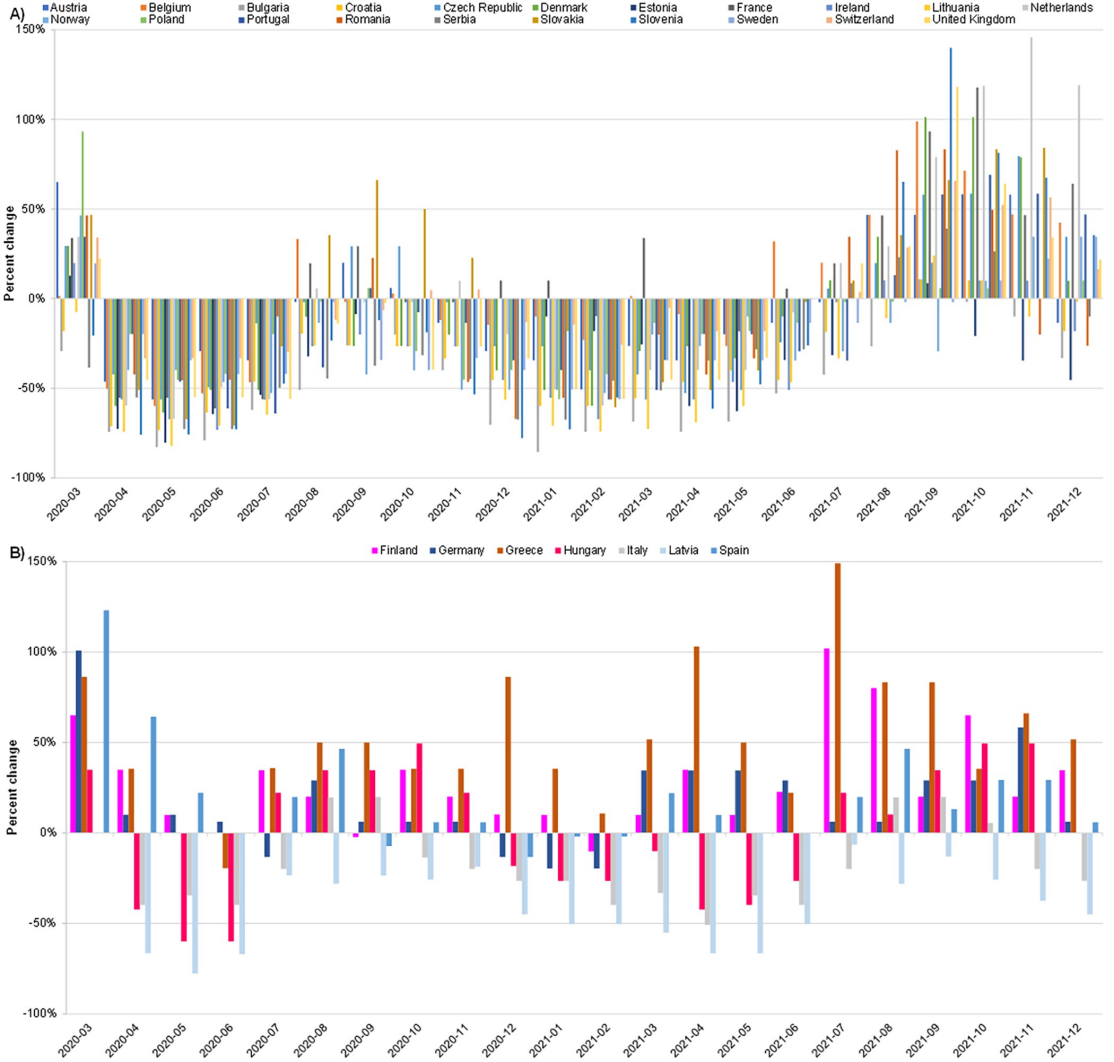
Percentage change in monthly absolute search volumes for varicella keywords, March 2020 through December 2021, compared with average search volumes during respective months in 2018 and 2019 in countries A) without UVV and B) with UVV. Percentage change was calculated by comparing the monthly search volumes recorded from March 2020 to December 2021, with the 2-year average of search volumes recorded during respective months in 2018 and 2019.

With few exceptions, countries without UVV recorded lower absolute volumes of search queries for varicella keywords during the 15-month period of April 2020 through July 2021, compared with volumes recorded during the respective months of pre-pandemic years (i.e., the average of 2018 and 2019; Fig 6A). The absolute volume of search queries for varicella keywords also largely followed this pattern in Italy, Latvia, and Hungary, countries with UVV, where declines were recorded in most months from April 2020 through July 2021 (Fig 6B). However, other countries with UVV, namely Finland, Germany, Greece, and Spain, recorded similar or higher levels of search volumes for varicella keywords from April 2020 through July 2021 compared with volumes recorded in 2018 and 2019 (Fig 6B). After July 2021, most countries included in the study recorded higher volumes of search queries for varicella keywords compared with volumes recorded in respective months of pre-pandemic years (2018 and 2019). This rebound of search query volumes for varicella keywords after July 2021 was not observed in 2 countries with UVV, namely Latvia and Italy.

## Discussion

We evaluated 7 years of Google Trends data on search queries for varicella keywords in 28 European countries, finding that search queries displayed seasonal variation that corresponded closely to varicella incidence and that the magnitude and seasonality of queries was higher in countries without UVV than in countries with UVV. Although Google Trends data have previously been used to evaluate epidemiological trends in various diseases, including childhood infections [18–20], this study is one of few to use this methodology, an important tool to assess varicella dynamics in countries which lack surveillance systems. Notably, our study revealed that the magnitude of search queries for varicella keywords declined markedly in the first 15 months of the COVID-19 pandemic but rebounded thereafter, a pattern which was more pronounced in countries without UVV than those with UVV.

Our study findings suggest that trends in search queries could be a good proxy of trends in varicella infection rates. Both relative and absolute search query volumes for varicella keywords during the 5 years preceding the COVID-19 pandemic displayed seasonal variation in countries without UVV, peaking in the autumn or winter and in the spring. This pattern aligns with seasonal variation reported in studies of incident varicella infections and varicella-related hospitalizations from several European countries [1, 19, 23–29]. Using data from Bulgaria as a comparator, we quantitatively evaluated clinical varicella incidence with the Google Trends Index, finding an adjusted R^2^ value of 0.75. This finding is consistent with that of other studies comparing Google Trends with clinical varicella data from France, Estonia, Mexico, Thailand and Australia, which reported correlations ranging from 0.65 to 0.81 [19, 30, 31]. The high correlation between varicella incidence and Google Trends data observed in this and other studies further suggests that search query data could reasonably serve as a proxy for varicella infection rates.

The correspondence between Google Trends data and trends in varicella infection rates was further supported by our findings that the magnitude and seasonal variation of search queries for varicella keywords diminished markedly after the introduction of UVV in Finland, Italy, Spain, and, to a lesser extent, in Hungary. Notably, in Hungary, UVV was introduced in 2019, shortly before the COVID-19 pandemic began, limiting the period of observation to evaluate the effect of UVV under pre-pandemic conditions. This aside, our findings accord with studies from Europe and the United States reporting declines in varicella infections and hospitalization in the years following UVV implementation [6, 7, 25, 32]. For example, in Germany UVV led to a greater than 50% reduction in varicella cases and a greater than 70% reduction in varicella-associated hospitalizations [7], while in Veneto, Italy, incident varicella in children fell by 90% following the introduction of a quadrivalent measles-mumps-rubella-varicella vaccine [32]. In the United States, routine varicella vaccination led to an overall 97% decline in incident cases from 1996 to 2014, including an 89% decline in cases among infants too young to be vaccinated; notably, declines in varicella incidence intensified following a switch from a 1- to a 2-dose regimen in 2006 [33–35].

We identified 1 study that evaluated the effect of UVV on trends in search queries for varicella keywords which found, as we did, that in countries with UVV, search query intensity displayed moderate to little seasonal variation [19]. This trend may be explained by the fact that UVV suppresses the transmission and seasonal variation of varicella infections [6, 7, 19, 25], which subsequently reduces information seeking on varicella.

Our study revealed that, in most countries, the onset of the COVID-19 pandemic on March 11, 2020 [21] disrupted normal seasonal variation in search query volumes for varicella keywords, which remained low through July 2021, before rebounding in August 2021. Notably, April 2020 through July 2021 was a period in which some European countries implemented strict infection control measures, such as lockdowns, school closings, social distancing, and mandatory mask wearing [13, 36]. These measures limited the spread of SARS-CoV-2, but also likely suppressed transmission of other infections. Several clinical studies have reported that the incidence of pediatric infections, including respiratory syncytial virus, gastroenteritis, bronchiolitis, and varicella, were substantially lower during the COVID-19 pandemic compared with the pre-pandemic period [12–14].

Notably, 1 study from France reported a 90% decline in varicella incidence alongside a substantial reduction in search query volumes for varicella keywords from March through December 2020 [13], a finding which aligns with that of our study. However, we followed trends in varicella search queries through December 2021, observing a rebound in search query volumes after the relaxation of COVID-19 infection control measures in July 2021 [36]. This observation highlights the potential for incurring an ‘immunity debt’, as decreased varicella transmission lowers herd immunity and could subsequently raise post-pandemic varicella incidence [12]. As varicella infection typically occurs in early childhood, in the absence of UVV, reduced herd immunity may delay varicella infection into adolescence or adulthood, during which varicella infections are associated with more severe outcomes [37]. Widespread vaccine-induced herd immunity to varicella may mitigate the effects of the ‘immunity debt’, as suggested by our finding that search query rebound was less pronounced in countries with UVV compared with those without UVV. Nevertheless, the COVID-19 pandemic lowered routine childhood vaccination coverage [12], which, in the absence of catch-up campaigns, may increase post-pandemic incidence of varicella, even in countries with UVV, with attendant clinical and financial consequences [38, 39].

Varicella poses a substantial socioeconomic and quality of life burden, which is currently under-ascertained by conventional approaches to varicella surveillance in many European countries [8, 10, 37, 40]. Given the close correspondence between trends in search query intensity and those of varicella incidence, search query data may be useful in buttressing conventional varicella surveillance systems or assessing the burden of varicella where surveillance data is absent. Notably, automated analyses of trends in internet searches for influenza keywords have been used to enhance influenza surveillance systems that are in development or in use in Europe, the United States, and Taiwan [20, 41–43]. Consideration should be given to using a similar approach to enhance the estimation of varicella incidence, in order to support the development of good surveillance systems, which are a fundamental component of resilient public health ecosystems.

This study has limitations. Although search query data correlated well with the observed trends in varicella incidence, such data do not equate to varicella case counts, as they may reflect health information seeking rather than the actual occurrence of illness. Notably, web search behavior may be influenced by media coverage, potentially distorting the relationship between varicella incidence and search query parameters. However, this potential bias was likely minimal in our study, as strong deviations from the usual seasonal trends in search intensity were not observed during pre-pandemic years. In addition, bias arising from missing data may affect analyses of search query trends, particularly when data series are composed of single-day values from small geographic units. In the current study, data comprised a time series of monthly averaged, primarily country-level values that did have any missing data points. Multiple searches for varicella keywords conducted by the same individual may also have inflated estimates derived from Google Trends data. To address this issue, the Google Trends algorithm eliminates repeated searches conducted by the same user within a short period of time, minimizing the influence of repeat searches on query data. Our study did not capture search activities for varicella symptoms, such as “itchy blisters” or “red rash,” and thus may have underestimated varicella-related search query volumes. Nevertheless, keywords were selected to reflect the most commonly used local language terms for varicella and were validated using country-specific internet searches; this approach has been used in other studies evaluating internet search data as a proxy for varicella incidence [13]. We considered March 2020 as a pre-pandemic month; however, COVID-19 transmission was underway before March 2020, and thus our analysis may have underestimated the early impact of COVID-19 on search query intensity.

In conclusion, this study demonstrated the potential of internet search query data to evaluate trends in varicella incidence in Europe, which declined in magnitude and seasonal variation following the introduction of UVV. This and other studies suggest that varicella incidence decreased and subsequently rebounded in response to COVID-19 pandemic interventions. Evaluation of search query data may be useful to assess changes in varicella incidence over seasons as well as following major events, such as the introduction of UVV and the COVID-19 pandemic, particularly in countries that lack robust varicella surveillance.

## Supporting information

S1 Table Uniform resource locator information of search queries for varicella keywords in European countries, 2015 to 2021. (DOCX)

S1 Fig Monthly relative search query popularity of varicella keywords, 2015 to 2021, in 19 countries or regions without UVV. (DOCX)

S2 Fig Monthly relative search query popularity of varicella keywords, 2015 to 2021, in countries where UVV was introduced before 2010: Germany, Greece, and Latvia. (DOCX)

S3 Fig A validation model of Google trends data and reported cases from Bulgaria, January 2015 through December 2021. (DOCX)

S4 Fig Monthly absolute search volumes for varicella keywords, 2018 to 2021. (DOCX)

## References

[1] Meszner Z, Wysocki J, Richter D, Zavadska D, Ivaskeviciene I, Usonis V, et al. Burden of varicella in Central and Eastern Europe: findings from a systematic literature review. Expert Rev Vaccines. 2019;18(3):281–93. doi: 10.1080/14760584.2019.1573145 . 30810402

[2] Lamont RF, Sobel JD, Carrington D, Mazaki-Tovi S, Kusanovic JP, Vaisbuch E, et al. Varicella-zoster virus (chickenpox) infection in pregnancy. BJOG. 2011;118(10):1155–62. doi: 10.1111/j.1471-0528.2011.02983.x ; PubMed Central PMCID: PMC3155623. 21585641 PMC3155623

[3] Glynn JR, Moss PAH. Systematic analysis of infectious disease outcomes by age shows lowest severity in school-age children. Sci Data. 2020;7(1):329. doi: 10.1038/s41597-020-00668-y ; PubMed Central PMCID: PMC7566589. 33057040 PMC7566589

[4] Poletti P, Melegaro A, Ajelli M, Del Fava E, Guzzetta G, Faustini L, et al. Perspectives on the impact of varicella immunization on herpes zoster. A model-based evaluation from three European countries. PLoS One. 2013;8(4):e60732. doi: 10.1371/journal.pone.0060732 ; PubMed Central PMCID: PMC3629254. 23613740 PMC3629254

[5] Marin M, Marti M, Kambhampati A, Jeram SM, Seward JF. Global Varicella Vaccine Effectiveness: A Meta-analysis. Pediatrics. 2016;137(3):e20153741. doi: 10.1542/peds.2015-3741 . 26908671

[6] Kauffmann F, Bechini A, Bonanni P, Casabona G, Wutzler P. Varicella vaccination in Italy and Germany—different routes to success: a systematic review. Expert Rev Vaccines. 2020;19(9):843–69. doi: 10.1080/14760584.2020.1825947 . 32969747

[7] Spoulou V, Alain S, Gabutti G, Giaquinto C, Liese J, Martinon-Torres F, et al. Implementing Universal Varicella Vaccination in Europe: The Path Forward. Pediatr Infect Dis J. 2019;38(2):181–8. doi: 10.1097/INF.0000000000002233 . 30408002

[8] Bollaerts K, Riera-Montes M, Heininger U, Hens N, Souverain A, Verstraeten T, et al. A systematic review of varicella seroprevalence in European countries before universal childhood immunization: deriving incidence from seroprevalence data. Epidemiol Infect. 2017;145(13):2666–77. doi: 10.1017/S0950268817001546 ; PubMed Central PMCID: PMC5647669. 28826422 PMC5647669

[9] World Health Organization (WHO). Varicella and herpes zoster vaccines: WHO position paper, June 2014—Recommendations. Vaccine. 2016;34(2):198–9. doi: 10.1016/j.vaccine.2014.07.068 . 26723191

[10] Riera-Montes M, Bollaerts K, Heininger U, Hens N, Gabutti G, Gil A, et al. Estimation of the burden of varicella in Europe before the introduction of universal childhood immunization. BMC Infect Dis. 2017;17(1):353. doi: 10.1186/s12879-017-2445-2 ; PubMed Central PMCID: PMC5437534. 28521810 PMC5437534

[11] Fefferman NH, Naumova EN. Dangers of vaccine refusal near the herd immunity threshold: a modelling study. Lancet Infect Dis. 2015;15(8):922–6. doi: 10.1016/S1473-3099(15)00053-5 . 25981883

[12] Cohen R, Ashman M, Taha MK, Varon E, Angoulvant F, Levy C, et al. Pediatric Infectious Disease Group (GPIP) position paper on the immune debt of the COVID-19 pandemic in childhood, how can we fill the immunity gap? Infect Dis Now. 2021;51(5):418–23. doi: 10.1016/j.idnow.2021.05.004 ; PubMed Central PMCID: PMC8114587. 33991720 PMC8114587

[13] Launay T, Souty C, Vilcu AM, Turbelin C, Blanchon T, Guerrisi C, et al. Common communicable diseases in the general population in France during the COVID-19 pandemic. PLoS One. 2021;16(10):e0258391. doi: 10.1371/journal.pone.0258391 ; PubMed Central PMCID: PMC8504745 interests: ML and IB are employees of IQVIA France. This does not alter our adherence to PLOS ONE policies on sharing data and materials. There are no patents, products in development or marketed products associated with this research to declare. 34634090 PMC8504745

[14] Toelen J, Ritz N, de Winter JP. Changes in pediatric infections during the COVID-19 pandemic: ’a quarantrend for coronials’? Eur J Pediatr. 2021;180(6):1965–7. doi: 10.1007/s00431-021-03986-4 ; PubMed Central PMCID: PMC7920544. 33649911 PMC7920544

[15] Brownstein JS, Freifeld CC, Madoff LC. Digital disease detection—harnessing the Web for public health surveillance. N Engl J Med. 2009;360(21):2153–5, 7. doi: 10.1056/NEJMp0900702 ; PubMed Central PMCID: PMC2917042. 19423867 PMC2917042

[16] Google Trends. Available from: https://www.google.com/trends.

[17] Google Ads. Available from: https://ads.google.com/intl/en_GM/home/tools/keyword-planner/.

[18] Cervellin G, Comelli I, Lippi G. Is Google Trends a reliable tool for digital epidemiology? Insights from different clinical settings. J Epidemiol Glob Health. 2017;7(3):185–9. doi: 10.1016/j.jegh.2017.06.001 ; PubMed Central PMCID: PMC7320449. 28756828 PMC7320449

[19] Bakker KM, Martinez-Bakker ME, Helm B, Stevenson TJ. Digital epidemiology reveals global childhood disease seasonality and the effects of immunization. Proc Natl Acad Sci U S A. 2016;113(24):6689–94. doi: 10.1073/pnas.1523941113 ; PubMed Central PMCID: PMC4914188. 27247405 PMC4914188

[20] Ginsberg J, Mohebbi MH, Patel RS, Brammer L, Smolinski MS, Brilliant L. Detecting influenza epidemics using search engine query data. Nature. 2009;457(7232):1012–4. doi: 10.1038/nature07634 . 19020500

[21] Cucinotta D, Vanelli M. WHO Declares COVID-19 a Pandemic. Acta Biomed. 2020;91(1):157–60. doi: 10.23750/abm.v91i1.9397 ; PubMed Central PMCID: PMC7569573. 32191675 PMC7569573

[22] Ministry of Health of the Republic of Bulgaria. National Center for Infectious and Parasitic Diseases 2022 [updated 2022July 15, 2022]. Available from: https://www.ncipd.org/index.php?option=com_content&view=featured&Itemid=730&lang=en.

[23] Bakasun V, Pahor D. Epidemiological Patterns of Varicella in the Period of 1977 to 2012 in the Rijeka District, Croatia. Epidemiology Research International. 2014. 10.1155/2014/193678.

[24] Widgren K. The Epidemiology of Varicella Zoster Virus Disease in Sweden—Before and After Vaccination Karolinksa Institutet; 2021.

[25] Piazza MF, Amicizia D, Paganino C, Marchini F, Astengo M, Grammatico F, et al. Has Clinical and Epidemiological Varicella Burden Changed over Time in Children? Overview on Hospitalizations, Comorbidities and Costs from 2010 to 2017 in Italy. Vaccines (Basel). 2021;9(12). doi: 10.3390/vaccines9121485 ; PubMed Central PMCID: PMC8705975. 34960231 PMC8705975

[26] Todorova TT. Seasonal dynamics of varicella incidence in Bulgaria. Future Virology. 2020;15(7). doi: 10.2217/fvl-2020-0012

[27] Socan M, Berginc N, Lajovic J. Varicella susceptibility and transmission dynamics in Slovenia. BMC Public Health. 2010;10:360. doi: 10.1186/1471-2458-10-360 ; PubMed Central PMCID: PMC2901375. 20573202 PMC2901375

[28] Vilibic-Cavlek T, Ljubin-Sternak S, Kolaric B, Kaic B, Sviben M, Kos L, et al. Immunity to varicella-zoster virus in Croatian women of reproductive age targeted for serology testing. Arch Gynecol Obstet. 2012;286(4):901–4. doi: 10.1007/s00404-012-2398-z . 22678561

[29] Arama V, Rafila A, Streinu-Cercel A, Pistol A, Bacruban R, Sandu R, et al. Varicella in Romania: epidemiological trends, 1986–2004. Euro Surveill. 2005;10(8):E050811 6. doi: 10.2807/esw.10.32.02775-en . 16785684

[30] Pelat C, Turbelin C, Bar-Hen A, Flahault A, Valleron A. More diseases tracked by using Google Trends. Emerg Infect Dis. 2009;15(8):1327–8. doi: 10.3201/eid1508.090299 ; PubMed Central PMCID: PMC2815981. 19751610 PMC2815981

[31] Milinovich GJ, Avril SM, Clements AC, Brownstein JS, Tong S, Hu W. Using internet search queries for infectious disease surveillance: screening diseases for suitability. BMC Infect Dis. 2014;14:690. doi: 10.1186/s12879-014-0690-1 ; PubMed Central PMCID: PMC4300155. 25551277 PMC4300155

[32] Giaquinto C, Gabutti G, Baldo V, Villa M, Tramontan L, Raccanello N, et al. Impact of a vaccination programme in children vaccinated with ProQuad, and ProQuad-specific effectiveness against varicella in the Veneto region of Italy. BMC Infect Dis. 2018;18(1):103. doi: 10.1186/s12879-018-3017-9 ; PubMed Central PMCID: PMC5839017. 29506477 PMC5839017

[33] Chaves SS, Lopez AS, Watson TL, Civen R, Watson B, Mascola L, et al. Varicella in infants after implementation of the US varicella vaccination program. Pediatrics. 2011;128(6):1071–7. doi: 10.1542/peds.2011-0017 . 22123875

[34] Lopez AS, Zhang J, Marin M. Epidemiology of Varicella During the 2-Dose Varicella Vaccination Program—United States, 2005–2014. MMWR Morb Mortal Wkly Rep. 2016;65(34):902–5. doi: 10.15585/mmwr.mm6534a4 . 27584717

[35] Kattan JA, Sosa LE, Bohnwagner HD, Hadler JL. Impact of 2-dose vaccination on varicella epidemiology: Connecticut—2005-2008. J Infect Dis. 2011;203(4):509–12. doi: 10.1093/infdis/jiq081 ; PubMed Central PMCID: PMC3071238. 21199882 PMC3071238

[36] If Government. Timeline of UK Government coronavirus lockdowns and measures, March 2020 to December 2021. 2022.

[37] Helmuth IG, Poulsen A, Suppli CH, Molbak K. Varicella in Europe-A review of the epidemiology and experience with vaccination. Vaccine. 2015;33(21):2406–13. doi: 10.1016/j.vaccine.2015.03.055 . 25839105

[38] Pawaskar M, Meroc E, Samant S, Flem E, Bencina G, Riera-Montes M, et al. Economic burden of varicella in Europe in the absence of universal varicella vaccination. BMC Public Health. 2021;21(1):2312. doi: 10.1186/s12889-021-12343-x ; PubMed Central PMCID: PMC8690977. 34930179 PMC8690977

[39] Thomas SL, Minassian C, Ganesan V, Langan SM, Smeeth L. Chickenpox and risk of stroke: a self-controlled case series analysis. Clin Infect Dis. 2014;58(1):61–8. doi: 10.1093/cid/cit659 ; PubMed Central PMCID: PMC3864501. 24092802 PMC3864501

[40] Jurkovic L, Katic M, Ozvacic Z, Stojanovic-Spehar S, Vinter-Repalust N. [Underestimation of varicella incidence—frequency of requests for medical assistance]. Acta Med Croatica. 2003;57(2):117–22. . 12879691

[41] Lu FS, Hattab MW, Clemente CL, Biggerstaff M, Santillana M. Improved state-level influenza nowcasting in the United States leveraging Internet-based data and network approaches. Nat Commun. 2019;10(1):147. doi: 10.1038/s41467-018-08082-0 ; PubMed Central PMCID: PMC6329822. 30635558 PMC6329822

[42] Hulth A, Rydevik G. GET WELL: an automated surveillance system for gaining new epidemiological knowledge. BMC Public Health. 2011;11:252. doi: 10.1186/1471-2458-11-252 ; PubMed Central PMCID: PMC3098167. 21510860 PMC3098167

[43] Chang YW, Chiang WL, Wang WH, Lin CY, Hung LC, Tsai YC, et al. Google Trends-based non-English language query data and epidemic diseases: a cross-sectional study of the popular search behaviour in Taiwan. BMJ Open. 2020;10(7):e034156. doi: 10.1136/bmjopen-2019-034156 ; PubMed Central PMCID: PMC7337886. 32624467 PMC7337886

