# Supplementary material for: Impact assessment of immunization and the COVID-19 pandemic on varicella across Europe using digital epidemiology methods: A descriptive study
Source: PLoS One. 2023 Apr 12;18(4):e0283465. doi: 10.1371/journal.pone.0283465 (PMC10096188; doi:10.1371/journal.pone.0283465)
Supplement: S1 Table — (DOCX) [file pone.0283465.s001.docx]

**S1 Table. Uniform Resource Locator information of search queries for varicella keywords in European countries, 2015 to 2021**

| **Country** | ***Keyword*** | **Category** | **Type of data** | **URL** |
| --- | --- | --- | --- | --- |
| Austria | *Windpocken* | All categories | Web search | https://trends.google.com/trends/explore?date=2015-01-01%202020-12-31&geo=AT&q=Windpocken |
| Belgium | *Windpokken* | All categories | Web search | https://trends.google.com/trends/explore?date=2015-01-01%202021-12-31&geo=BE&q=Windpokken |
| Belgium | *Varicelle* | All categories | Web search | https://trends.google.com/trends/explore?date=2015-01-01%202021-12-31&geo=BE&q=Varicelle |
| Bulgaria | *Bарицела* | All categories | Web search | https://trends.google.com/trends/explore?date=2015-01-01%202021-12-31&geo=BG&q=B%D0%B0%D1%80%D0%B8%D1%86%D0%B5%D0%BB%D0%B0 |
| Croatia | *Vodene kozice* | All categories | Web search | https://trends.google.com/trends/explore?date=2015-01-01%202021-12-31&geo=HR&q=Vodene%20kozice |
| Czech Republic | *Neštovice* | All categories | Web search | https://trends.google.com/trends/explore?date=2015-01-01%202021-12-31&geo=CZ&q=Ne%C5%A1tovice |
| Denmark | *Skoldkopper* | All categories | Web search | https://trends.google.com/trends/explore?date=2015-01-01%202021-12-31&geo=DK&q=Skoldkopper |
| Estonia | *Tuulerõuged* | All categories | Web search | https://trends.google.com/trends/explore?date=2015-01-01%202021-12-31&geo=EE&q=Tuuler%C3%B5uged |
| Finland | *Vesirokko* | All categories | Web search | https://trends.google.com/trends/explore?date=2015-01-01%202021-12-31&geo=FI&q=Vesirokko |
| France | *Varicelle* | All categories | Web search | https://trends.google.com/trends/explore?date=2015-01-01%202021-12-31&geo=FR&q=Varicelle |
| Germany | *Windpocken* | All categories | Web search | https://trends.google.com/trends/explore?date=2015-01-01%202021-12-31&geo=DE&q=Windpocken |
| Greece | *ανεμοβλογιά* | All categories | Web search | https://trends.google.com/trends/explore?date=2015-01-01%202021-12-31&geo=GR&q=%CE%B1%CE%BD%CE%B5%CE%BC%CE%BF%CE%B2%CE%BB%CE%BF%CE%B3%CE%B9%CE%AC |
| Hungary | *Bárányhimlő* | All categories | Web search | https://trends.google.com/trends/explore?date=2015-01-01%202021-12-31&geo=HU&q=B%C3%A1r%C3%A1nyhiml%C5%91 |
| Ireland | *Chickenpox* | All categories | Web search | https://trends.google.com/trends/explore?date=2015-01-01%202021-12-31&geo=IE&q=Chickenpox |
| Italy | *Varicella* | All categories | Web search | https://trends.google.com/trends/explore?date=2015-01-01%202021-12-31&geo=IT&q=Varicella |
| Latvia | *Vējbakas* | All categories | Web search | https://trends.google.com/trends/explore?date=2015-01-01%202021-12-31&geo=LV&q=V%C4%93jbakas |
| Lithuania | *Vėjaraupiai* | All categories | Web search | https://trends.google.com/trends/explore?date=2015-01-01%202021-12-31&geo=LT&q=V%C4%97jaraupiai |
| Norway | *Vannkopper* | All categories | Web search | https://trends.google.com/trends/explore?date=2015-01-01%202021-12-31&geo=NO&q=Vannkopper |
| Poland | *Ospa* | All categories | Web search | https://trends.google.com/trends/explore?date=2015-01-01%202021-12-31&geo=PL&q=Ospa |
| Portugal | *Varicela* | All categories | Web search | https://trends.google.com/trends/explore?date=2015-01-01%202021-12-31&geo=PT&q=Varicela |
| Romania | *Varicelă* | All categories | Web search | https://trends.google.com/trends/explore?date=2015-01-01%202021-12-31&geo=RO&q=Varicel%C4%83 |
| Serbia | *Ovčije boginje* | All categories | Web search | https://trends.google.com/trends/explore?date=2015-01-01%202021-12-31&geo=RS&q=Ov%C4%8Dije%20boginje&hl |
| Slovakia | *Ovčie kiahne* | All categories | Web search | https://trends.google.com/trends/explore?date=2015-01-01%202021-12-31&geo=SK&q=Ov%C4%8Die%20kiahne&hl |
| Slovenia | *Norice* | All categories | Web search | https://trends.google.com/trends/explore?date=2015-01-01%202021-12-31&geo=SI&q=Norice |
| Spain | *Varicela* | All categories | Web search | https://trends.google.com/trends/explore?date=2015-01-01%202021-12-31&geo=ES&q=Varicela |
| Sweden | *Vattkoppor* | All categories | Web search | https://trends.google.com/trends/explore?date=2015-01-01%202021-12-31&geo=SE&q=Vattkoppor |
| Switzerland | *Windpocken* | All categories | Web search | https://trends.google.com/trends/explore?date=2015-01-01%202021-12-31&geo=CH&q=Windpocken |
| Switzerland | *Varizellen* | All categories | Web search | https://trends.google.com/trends/explore?date=2015-01-01%202021-12-31&geo=CH&q=Varizellen |
| Netherlands | *Waterpokken* | All categories | Web search | https://trends.google.com/trends/explore?date=2015-01-01%202021-12-31&geo=NL&q=Waterpokken |
| United Kingdom | *Chickenpox* | All categories | Web search | https://trends.google.com/trends/explore?date=2015-01-01%202021-12-31&geo=GB&q=Chickenpox |
