# Supplementary material for: Impact assessment of immunization and the COVID-19 pandemic on varicella across Europe using digital epidemiology methods: A descriptive study
Source: PLoS One. 2023 Apr 12;18(4):e0283465. doi: 10.1371/journal.pone.0283465 (PMC10096188; doi:10.1371/journal.pone.0283465)
Supplement: S1 Fig — (DOCX) [file pone.0283465.s002.docx]

**S1 Fig. Monthly relative search query popularity of varicella keywords, 2015 to 2021, in 19 countries or regions without UVV**

The Google Trends Index reflects the relative search query popularity. The gray area shows the search query popularity predicted in the absence of COVID-19, estimated as the 5-year average of Google Index values observed in the same calendar month, from 2015 through 2019.
