# Supplementary material for: Impact assessment of immunization and the COVID-19 pandemic on varicella across Europe using digital epidemiology methods: A descriptive study
Source: PLoS One. 2023 Apr 12;18(4):e0283465. doi: 10.1371/journal.pone.0283465 (PMC10096188; doi:10.1371/journal.pone.0283465)
Supplement: S2 Fig — (DOCX) [file pone.0283465.s003.docx]

**S2 Fig. Monthly relative search query popularity of varicella keywords, 2015 to 2021, in countries where UVV was introduced before 2010: Germany, Greece, and Latvia**
