# Supplementary material for: Impact assessment of immunization and the COVID-19 pandemic on varicella across Europe using digital epidemiology methods: A descriptive study
Source: PLoS One. 2023 Apr 12;18(4):e0283465. doi: 10.1371/journal.pone.0283465 (PMC10096188; doi:10.1371/journal.pone.0283465)
Supplement: S3 Fig — (DOCX) [file pone.0283465.s004.docx]

S3 Fig. A validation model of Google Trends data and reported cases from Bulgaria, January 2015 through December 2021

1. **Model parameters**

A univariate linear regression model was fitted to assess whether the search query data for varicella sourced from Google trends could be used as a potential index of the observed varicella diagnoses in Bulgaria. In this univariate linear model, monthly counts of varicella cases reported to the Bulgarian National Center of Infectious and Parasitic were regressed against Google Trends data. Daily Google searches were assumed to be independent of each other; seasonal parameters were not included in the model. Normality assumptions of the model were met. However, potential deviations from normality were not expected to impact the results due to the large sample size and large mean values. The model output is shown in the table below, and the equation created by the linear regression is shown in the figure below. The R^2^ index indicates that varicella Google trends could explain a fair part of the variability of the observed diagnosis (R^2^ index=74.8%). The p-value of the F test for the model fit was less than 0.05.

| Regression Statistics | |
| --- | --- |
| R^2^ | 0.7516 |
| Adjusted R^2^ | 0.7486 |

|  | Coefficients | Standard Error | P-value | Lower 95% | Upper 95% |
| --- | --- | --- | --- | --- | --- |
| Intercept | -268.5729 | 153.0151 | 0.0830 | -572.9688 | 35.8229 |
| Google Trends | 50.1216 | 3.1813 | <0.0001 | 43.7930 | 56.4501 |

**B) Scatter plot of observed versus predicted values**

**
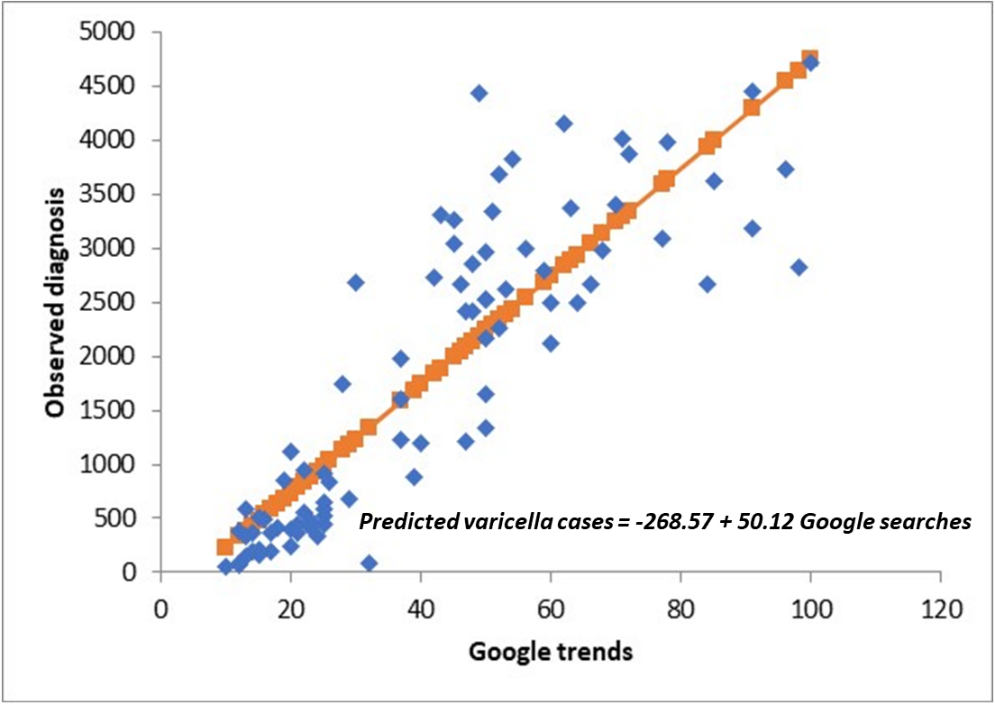
**

Data were output from a univariate model that regressed reported varicella cases against Google Trends data. The equation for the model was as follows: Reported cases = -268.57 + 50.12* Google searches; the P value of the model’s F-test was <0.05. Blue diamonds represent observed data points; the orange line represents predicted values.
