# Supplementary material for: Impact assessment of immunization and the COVID-19 pandemic on varicella across Europe using digital epidemiology methods: A descriptive study
Source: PLoS One. 2023 Apr 12;18(4):e0283465. doi: 10.1371/journal.pone.0283465 (PMC10096188; doi:10.1371/journal.pone.0283465)
Supplement: S4 Fig — (DOCX) [file pone.0283465.s005.docx]

**S4 Fig. Monthly absolute search volumes for varicella keywords, 2018 to 2021**

The maximum value of the vertical axis was set to 250,000 in the graphs of France, Germany, Italy, the Netherlands, Poland, Sweden, and the United Kingdom, as these countries had a high monthly volumes of search queries; in the graphs of all other countries the maximum value of the vertical axis was set to 50,000. The double bars in graphs for Belgium and Switzerland reflect the fact that these countries have two different lingual regions.
